# Evaluation of threshold selection methods for adaptive kernel density estimation in disease mapping

**DOI:** 10.1186/s12942-018-0129-9

**Published:** 2018-05-08

**Authors:** Warangkana Ruckthongsook, Chetan Tiwari, Joseph R. Oppong, Prathiba Natesan

**Affiliations:** 1 Department of Biological Sciences, University of North Texas, Denton, TX USA; 2 Department of Geography and the Environment, University of North Texas, Denton, TX USA; 3 Department of Educational Psychology, University of North Texas, Denton, TX USA

**Keywords:** Kernel density estimation, Bandwidth selection, Threshold, Monte Carlo simulation, Disease mapping

## Abstract

**Background:**

Maps of disease rates produced without careful consideration of the underlying population distribution may be unreliable due to the well-known small numbers problem. Smoothing methods such as Kernel Density Estimation (KDE) are employed to control the population basis of spatial support used to calculate each disease rate. The degree of smoothing is controlled by a user-defined parameter (bandwidth or threshold) which influences the resolution of the disease map and the reliability of the computed rates. Methods for automatically selecting a smoothing parameter such as normal scale, plug-in, and smoothed cross validation bandwidth selectors have been proposed for use with non-spatial data, but their relative utilities remain unknown. This study assesses the relative performance of these methods in terms of resolution and reliability for disease mapping.

**Results:**

Using a simulated dataset of heart disease mortality among males aged 35 years and older in Texas, we assess methods for automatically selecting a smoothing parameter. Our results show that while all parameter choices accurately estimate the overall state rates, they vary in terms of the degree of spatial resolution. Further, parameter choices resulting in desirable characteristics for one sub group of the population (e.g., a specific age-group) may not necessarily be appropriate for other groups.

**Conclusion:**

We show that the appropriate threshold value depends on the characteristics of the data, and that bandwidth selector algorithms can be used to guide such decisions about mapping parameters. An unguided choice may produce maps that distort the balance of resolution and statistical reliability.

## Background

Disease maps, an essential component of epidemiological surveillance, are used to illustrate the geographic distribution of diseases. Disease outcomes are typically represented as rates, which are computed by dividing the number of disease cases by the population contained within some geographic region such as a zip code or county. Rates that are computed without careful consideration of the underlying population distribution may be unreliable due to the well-known small numbers problem [1]. For example, areas with small populations are more likely to produce unstable rate estimates compared to areas with larger population sizes. Smoothing methods including kernel density estimation are commonly used to address the problem of unstable rates [1–11].

Kernel Density Estimation (KDE) is a non-parametric method that can be used to explore the spatial density of point data [1]. In the context of disease mapping, KDE methods operate by computing rates within a moving spatial window or kernel (typically a circle) placed across the entire study area. A ratio of the density of events (i.e., cases) and the density of the background (i.e., the population) is calculated within each kernel [12]. Another KDE method computes the rate by dividing the number of cases that fall inside a kernel by the population that is contained within the same kernel [4, 9].

The size of the kernel, bandwidth, is a crucial parameter that influences the degree of smoothing on the map in KDE [13–15]. The bandwidth can be either fixed or variable (adaptive). For the fixed bandwidth approach, the kernel has a fixed-size radius, and all kernels (circles) have the same radii. In health studies, the fixed bandwidth approach may not be suitable since populations are not evenly distributed across geographic space. Moreover, unstable rates may result if the circle falls in low population-density areas. Similarly, in the adaptive bandwidth approach, the kernel radius grows or shrinks to accommodate varying population sizes. The minimum population size that is used to define the kernel bandwidth, and consequently the degree of smoothing on a map, is a user-defined parameter. We will refer to this as the threshold value (*h*).

**Fig. 1:**
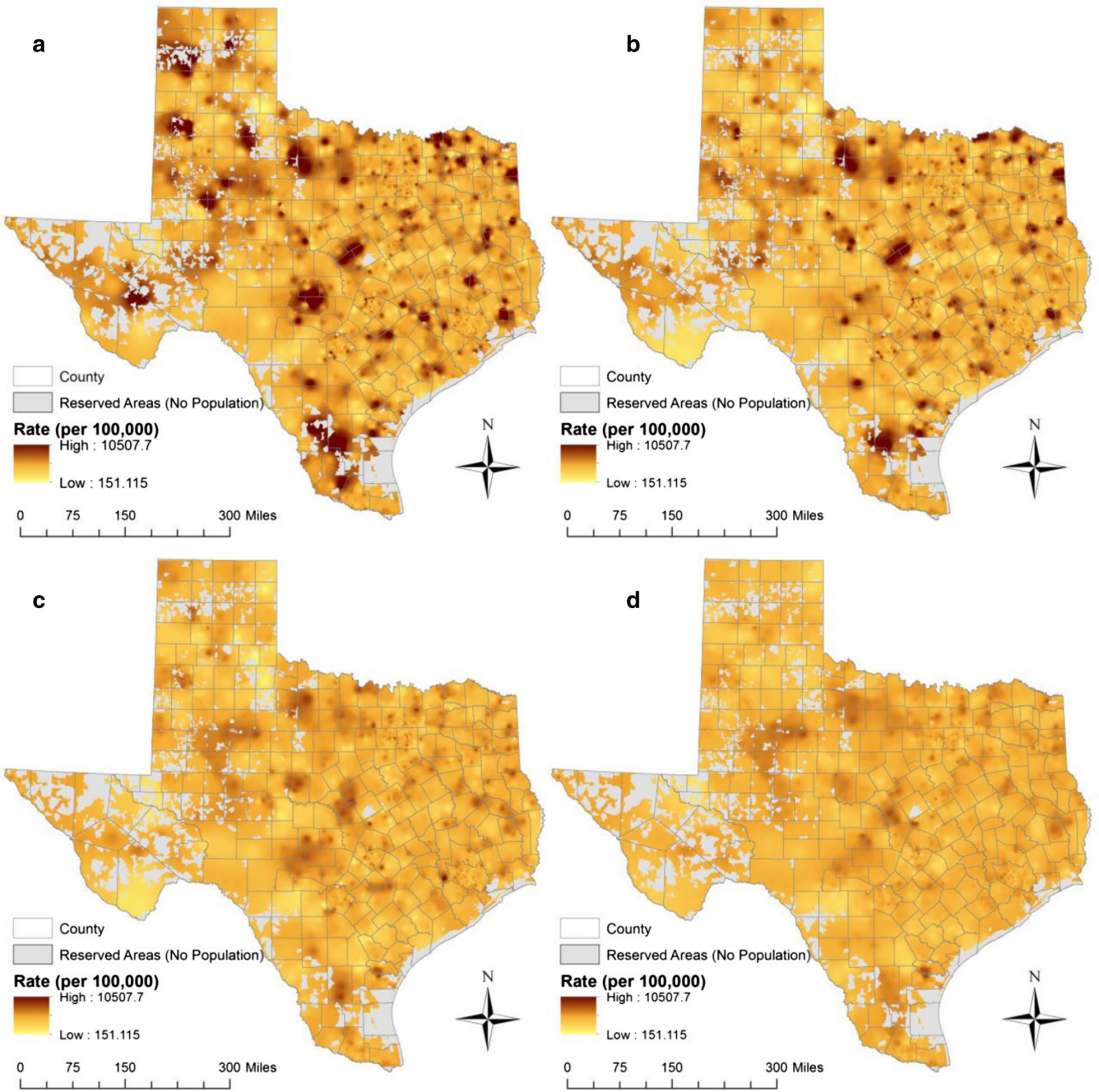
Geographic distribution of age-specific heart disease mortality rates for males aged 65 years and older, 2009–2013. Maps were created using the adaptive bandwidth kernel density estimation method with various bandwidths (*h*): **a** *h* = 50; **b** *h* = 100; **c** *h* = 500; **d** *h* = 1000. (Note: the data were obtained from CDC NCHS [16])

Figure 1 illustrates the spatial distribution of heart disease mortality rates for males aged 65 years and older using data obtained from the Centers for Disease Control and Prevention (CDC), National Center for Health Statistics (NCHS) [16]. We produced this map using the adaptive kernel density estimation method with different threshold values. As shown in Fig. 1a, when using the smallest threshold value (*h* = 50), the resulting map portrays high levels of geographic detail in the estimated rate. However, as the thresholds increase, the resulting maps show lower levels of geographic detail (Fig. 1b–d). Further, maps produced using small threshold values tend to display greater fluctuations in rate estimates (*µ* = 1330 per 100,000 population, *σ* = 639.9 at *h* = 50). In contrast, maps produced using larger threshold values tend to show lower levels of fluctuation (*µ* = 1209.5 per 100,000 population, *σ* = 268.4 at *h* = 1000). The trade-off between geographic detail and reliability depends on the choice of the threshold value. A value that is too small may result in under-smoothing, i.e., high levels of geographic detail but greater fluctuation in rate estimates (Fig. 1a). Conversely, a value that is too large will result in over-smoothing, i.e., low levels of geographic detail but less fluctuation in rate estimates (Fig. 1d).

The problem of choosing an appropriate smoothing parameter—bandwidth or threshold—has been discussed in previous studies [1, 4–6, 9, 11–13, 17–20]. Silverman [13] and Wand and Jones [20] recommend subjective selection of the bandwidth parameter based on visual inspection. The process of visual evaluation of the bandwidth parameter begins with examining several plots of the data and selecting the density that is the “most pleasing” in some sense [20:58]. Although this approach has been used by others [12:654], the process can be time-consuming if many density estimates are required. In other cases, mapmakers may not utilize information about the structure of the data to inform choice of threshold value.

Many bandwidth selectors available for use with non-spatial data could potentially be adapted for spatial data. However, their applicability for the purposes of creating disease maps has not been evaluated. Non-spatial bandwidth selectors may be grouped into two classes—(a) quick and simple, and (b) hi-tech bandwidth selectors [20]. Quick and simple bandwidth selectors aim to find a threshold value that is reasonable for a wide range of data distributions. One such method is the normal scale bandwidth selector [20]. This method recommends a bandwidth value which can be used as a starting point or a “first guess” [20]. The bandwidth is calculated by referencing a standard distribution that is derived from the data (see Silverman [13] and Wand and Jones [20] for details). In contrast, hi-tech bandwidth selectors, which are data-driven, seek to find an optimal bandwidth by minimizing the mean integrated square error (MISE) of the kernel density estimator [20, 21]. For example, plug-in [22] and smoothed cross-validation [23] bandwidth selectors estimate a pre-smoothing parameter based on the pairwise differences of the observations obtained using the pilot bandwidth value. The pre-smoothing parameter is then used to find the optimal bandwidth value [20, 25]. These two approaches are highly rated for bandwidth convergence and statistical performance [23, 24]. Additional information on the theoretical basis of these methods are available in Silverman [13], Wand and Jones [20], Chiu [21], Wand and Jones [22], Hall and Marron [23], Hall et al. [26].

In summary, while automatic bandwidth selections methods have been used to produce distributions of non-spatial data, their suitability for threshold selection in disease mapping remains unknown. In this study, we use a simulated dataset on heart disease mortality to examine applicability of applying commonly used automated bandwidth selection methods to determine optimal threshold values with the objective of producing disease maps with high levels of geographic details and statistical reliability.

## Methods

**Fig. 2:**
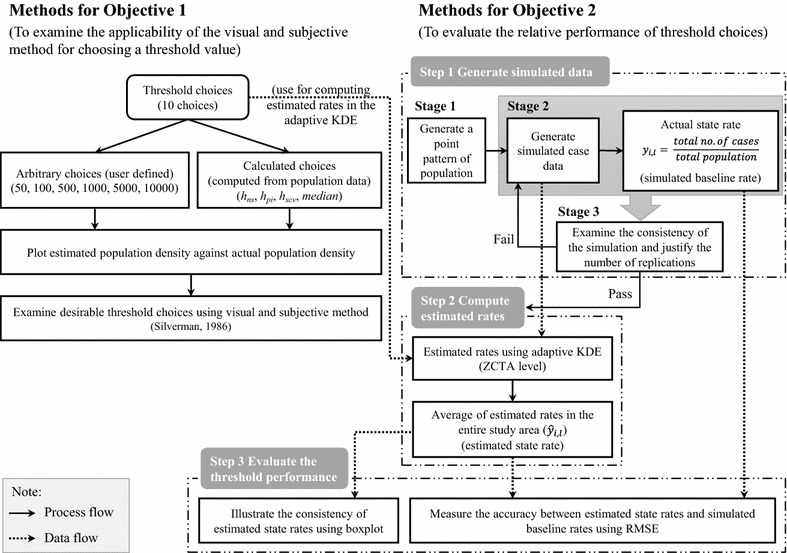
Flow chart of methodology showing the steps using in this study

The methods used in this study are presented in two parts (Fig. 2). First, we examine the applicability of the visual and subjective methods for choosing a threshold value (Objective 1). Initially, since we assume that mapmakers will select threshold values based on arbitrary choices or some knowledge of the disease, we use values ranging from 50 to 10,000. Subsequently, we use bandwidth selection methods—normal scale (*h*_*ns*_), plug-in (*h*_*pi*_), smoothed cross-validation (*h*_*scv*_), and *median*—for comparison. Our study uses the 10-year age-stratified population data for males in Texas obtained from the 2010 U.S. Census Bureau at the zip code level (Table 1) [27].

**Table 1:** Age-adjusted and age-specific heart disease death rates for males in Texas by age group, 2009–2013 [16], and population distribution from the 2010 U.S. Census Bureau [27]

Age	Population	Range of aggregated population at the ZCTA level	Rate (per 100,000)
35–44	1,722,904	[1, 7925]	33.87
45–54	1,702,639	[1, 7407]	115.15
55–64	1,256,976	[1, 4948]	297.36
65+	1,135,517	[1, 4792]	1245.93
Total (35+)	5,818,036	[1, 22,555]	351.15

Second, we evaluate the relative performance of different threshold values for disease maps using the same dataset (Objective 2). We first generated a Monte Carlo simulated dataset of age-specific heart disease mortality rates among males aged 35 years and older in Texas (Table 1). Statewide rates for generating the simulated case counts in each age group were obtained from the CDC NCHS [16]. Finally, maps produced using both approaches (Objectives 1 and 2) are compared.

### Methods for objective 1

A total of ten thresholds were used in this study. Six thresholds were a series of arbitrary choices—50, 100, 500, 1000, 5000, and 10,000. These six thresholds remained constant for all age groups. The remaining four were calculated based on population data aggregated at the zip code level using *median* and three bandwidth selectors—the normal scale (*h*_*ns*_), the plug-in (*h*_*pi*_), and the smoothed cross-validation (*h*_*scv*_). The *median* threshold was determined by computing the median population value across all zip codes. Threshold values from the three bandwidth selectors—*h*_*ns*_, *h*_*pi*_, and *h*_*scv*_—were computed using the ks-package in *R* [28]. Since these four thresholds were calculated based on data, their values varied among the age groups. We selected desirable threshold options using visual and subjective examination of the data as suggested by Silverman [13]. This involved generating plots of estimated population density against the actual population density. For each age group, estimated population densities were computed using a kernel function with each of the ten thresholds. The actual population density was generated from the population data using a gamma distribution. We chose the gamma distribution for two reasons: it mimics the population of the region by age group as shown in Table 1 with maximum number of people in the age group 35–44 and decreasing gradually to the age group 65+. Secondly, the gamma distribution does not allow for negative values in the distribution unlike say, a normal distribution where all real values are probable. It consists of two positive parameters—shape (*k*) and scale (θ) parameters. These two parameters were calculated using mean and standard deviation of the population data (Eqs. 1 and 2). This process was also performed in *R* using probability density function (Eq. 3).

1 $$k = \left( {\frac{\mu }{\sigma }} \right)^{2}$$

2 $$\theta = \frac{{\sigma^{2} }}{\mu }$$

3 $$f\left( x \right) = \frac{1}{{\theta^{k} \varGamma \left( k \right)}}x^{k - 1} e^{ - x/\theta }$$

where *k* and θ are shape and scale parameters respectively, *µ* and *σ* are respectively the mean and the standard deviation of the population, Γ(*k*) is the gamma function, and f(x) is the probability density function.

### Methods for objective 2

#### Step 1: Methods to generate simulated data

**Fig. 3:**
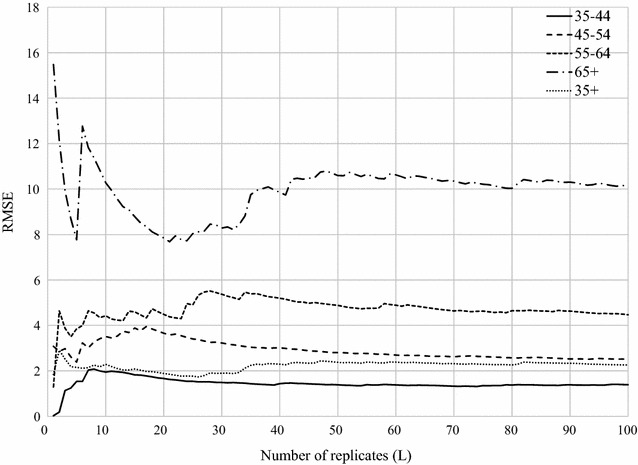
The running RMSE between the simulated baseline rates and the true value as a function of the number of replicates (*L*) for all age groups

The aim of this step was to generate simulated case data. This step comprised of three stages:

1. Generate a point pattern of male populations by age at the ZCTA level
  The data used in this process are (1) male population data stratified by age as shown in Table 1, and (2) a ZIP Code Tabulation Areas (ZCTAs) cartographic boundary file obtained from Topologically Integrated Geographic Encoding and Referencing (TIGER) [29]. Note that the ZCTAs are created by the U.S. Census Bureau and approximate the spatial boundaries of postal zip code service areas [30]. The age-stratified population data were joined to the ZCTA cartographic boundary file in ArcGIS 10.2. Then, the random point generation tool in ArcGIS 10.2 was used to create a point distribution, where each point (*x*_*i,s*_) represents a simulated individual in age group *i* residing in ZCTA *s.* The age group $i \in I\quad {\text{where}}\quad {\text{I = }}\left\{ { 3 5 { - 44, 45 - 54, 55 - 64, 65 + }} \right\}$.
2. Generate simulated cases from a point pattern of male populations obtained from stage 1To classify a simulated individual as a case, a random number was assigned to each point in the random point pattern generated from stage 1. The random number was generated from a uniform distribution on the interval (0, 1) under the assumption that each person has an equal probability of being designated as a case. The probability that a simulated point, *x*_*i,s*_, would be classified as a case (*c*_*i,s*_) was determined using observed age-specific heart disease mortality rates (Table 1). For example, the observed age-specific heart disease death rate for males aged 35–44 years old in Texas was 33.87 per 100,000 (0.0003387) (Table 1). If a random number generated was in the range 0.0000001 to 0.0003387, it was classified as a simulated case. This process was replicated 100 times to produce 100 different instances of the case distribution—i.e., a 100 simulated maps of heart disease mortality could be produced from this data. For each simulated dataset (*l*, where *l *= 1, 2, …, 100), state rates, called simulated baseline rates, were computed for each age group as well as for all-groups combined. The rate (*y*_*i,l*_) was computed using:
  4 $$y_{i,l} = \frac{{C_{i,l} }}{{P_{i} }}$$
  where *C*_*i,l*_ was the total number of simulated cases for age group *i* at the *l*th simulation, and *P*_*i*_ was the total population for age group *i*.
3. Examine the consistency of the simulation and justify the number of replications. For each age group, we examined the consistency of the simulated baseline rates ($y_{i,l}$) and justified the number of replications using a scatter plot of the running root mean squared error (RMSE^M^) against the total number of replications [31]. The RMSE is a function of the average difference between the simulated baseline rates and the true value, i.e., CDC’s heart disease mortality rate (Table 1) using the following formula:
  5 $$RMSE_{i,L}^{M} = \sqrt {\frac{1}{L}\mathop \sum \limits_{l = 1}^{L} \left( {y_{i,l} - Y_{i} } \right)^{2} }$$
  where *L* was the total number of replications, *y*_*i,l*_ was the simulated baseline rate of age group *i* at *l*th simulation, and *Y*_*i*_ was the true rate of age group *i*. Figure 3 illustrates that the magnitude of the difference between the simulated baseline rate and the true value (RMSE) stabilizes as *L* increases. In this study, when *L* > 50, the stable state was achieved for all age groups. In this study, we used 100 replications. Based on recommendations by Natesan [32], we also examined (1) the coverage rate, and (2) bias of interval estimates (Table 2). Coverage rate is defined as the percentage of statistical estimate intervals that contain the true values. Bias of interval estimates is computed as the percentage of the statistical estimate intervals that overestimate and underestimate the true value [32]. While the coverage rates for the 95% interval estimates are typically expected to be around 95%, our results show that they were extremely low—less than 20% for all age groups (Table 2). However, as the percentages of over- and under-estimates are more-or-less equal, we can conclude that the simulation was unbiased.

**Table 2:** Summaries of characteristics of simulated baseline rate distribution

Age group	Mean	SD	Coverage rate (%)	Over-estimated (%)	Under-estimated (%)
35–44	33.92	1.40	17	50.6	49.4
45–54	115.17	2.52	11	49.4	50.6
55–64	297.60	4.49	20	56.2	43.8
65+	1245.93	10.21	16	47.6	52.4
35+	351.12	2.27	14	52.3	47.7

#### Step 2 Methods to compute estimated rates

For each age group *i* at the *l*th simulation, estimated rates were computed using the KDE method with aggregated simulated cases as the numerators, and the population data as the denominators. The KDE method was applied to all ten threshold values. This process was performed using the Web-based Disease Mapping Analysis Program (WebDMAP) [33] and custom code written in Python. As a result, 100 estimated rates were produced for each threshold and for each age group *i*. These rates, which were obtained at the ZCTA level, were aggregated to the state level (called estimated state rate, $\widehat{y}_{i,l}$).

#### Step 3 Methods to evaluate threshold performance

To evaluate the relative performance of each threshold choice, the estimated state rates ($\widehat{y}_{i,l}$) resulting from different thresholds were compared to the simulated baseline rates in each age group *i* (*y*_*i,l*_ from Eq. 4). The root-mean-square-error (RMSE) was employed to measure the accuracy of the estimated state rates in estimating the simulated baseline rates using the following formula:

6 $$RMSE_{i,t} = \sqrt {\frac{1}{100}\mathop \sum \limits_{l = 1}^{100} \left( {\widehat{y}_{i,t,l} - y_{i,l} } \right)^{2} }$$

where RMSE_*i,t*_ was the RMSE of age group *i* and threshold *t*, $\widehat{y}_{i,t,l}$ was the estimated state rate of age group *i* and threshold *t* at the *l*th simulation, and $y_{i,l}$ was the simulated baseline rate of age group *i* at the *l*th simulation. Further, to illustrate the consistency of the rates computed from each threshold ($\widehat{y}_{i,t,l}$), a box-plot was generated to display the variation of 100 estimated state rates for each age group.

## Results and discussion

### The impact of threshold choice on population density estimates

The calculated thresholds for the three selectors—plug-in (*h*_*pi*_), smoothed cross-validation (*h*_*scv*_), normal scale (*h*_*ns*_)—and *median* are shown in Table 3. The *h*_*pi*_ and *h*_*scv*_ selectors result in the smallest threshold values. In contrast, the *h*_*ns*_ and *median* selectors are approximately 4 and 8 times larger, respectively for the age groups 55–64, 65 years and older, and the overall population (aged 35 years and older). Further, the *h*_*ns*_ and *median* selectors are also approximately 5 and 7 times larger for the two youngest groups—35 to 44 and 45 to 54. These results indicate that for the same data, different bandwidth selectors provide different threshold values. For this data, the *h*_*pi*_ and *h*_*scv*_ recommendations produce maps that provide greater geographic detail (lower levels of smoothing), but also larger fluctuations in estimated rates. Conversely, the other two bandwidth selectors produce greater levels of smoothing, but fewer fluctuations in rates.

**Table 3:** Descriptive results and calculated thresholds stratified by age group

Age groups	Total population	Range	No. of ZCTAs	Calculated thresholds				% ZCTAs with specified minimum population	
				*h*_*pi*_	*h*_*scv*_	*h*_*ns*_	Median	≤ 100 (%)	≤ 300 (%)
35–44	1,722,904	[1, 7925]	1911	53	56	280	327	32	48
45–54	1,702,639	[1, 7407]	1910	57	55	255	399	28	45
55–64	1,256,976	[1, 4948]	1906	44	41	177	342	30	48
65+	1,135,517	[1, 4792]	1902	41	40	156	330	28	48
Total (35 +)	5,818,036	[1, 22,555]	1920	200	189	837	1411	14	25

**Fig. 4:**
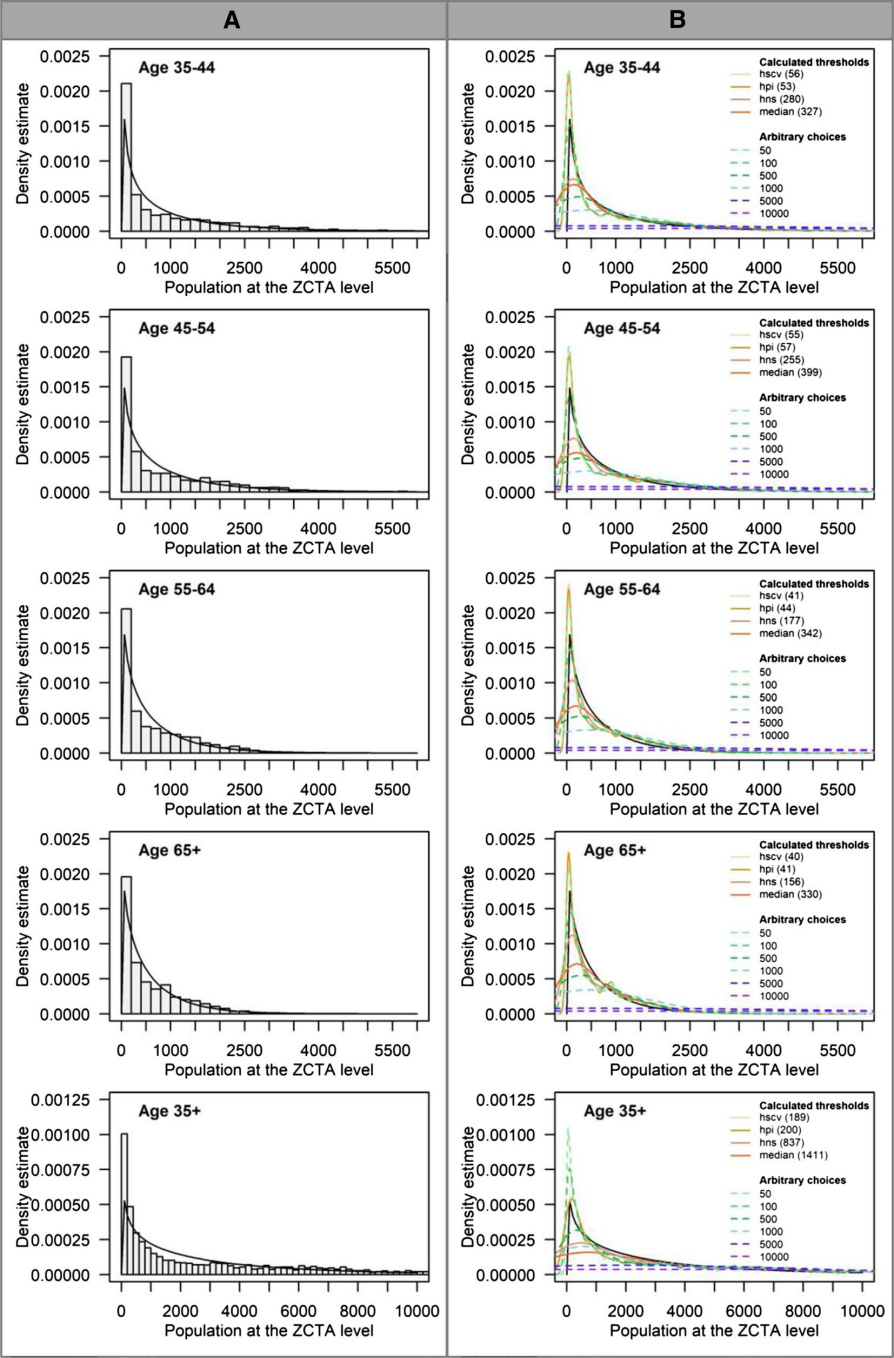
Density curves overlaid on population distribution (age > 35; ZCTA level). Column **A** describes the gamma distribution. Column **B** describes threshold choices

In Fig. 4, the density curves for populations obtained after applying each threshold (*h*_*pi*_, *h*_*scv*_, *h*_*ns*_, *median,* and six arbitrary choices—50, 100, 500, 1000, 5000, 10,000) are compared to the actual population distribution (see Methods—Objective 1). For each chart, the X-axis represents population with a bin size of 200 and the Y-axis is the density of ZCTAs.

The actual population density (Fig. 4 column A) tends to follow a gamma distribution (the black line) for all age groups, which indicates that the population is not evenly distributed. Thus, many ZCTAs have low populations, and the number of ZCTAs with large populations is small. This is indicated by a long tail to the right of the distribution. Figure 4 column B illustrates the population density estimates computed from all ten thresholds. For all age groups, the population density estimates computed from thresholds, *h* = 50, 100, *h*_*pi*_, and *h*_*scv*_, provide similar density curve characteristics. The density estimates have a sharp peak and closely match the actual gamma distribution. The resulting density curves from these four thresholds contain fluctuations at the tail end of the distribution. This suggests that these four thresholds may be too small for all age groups. For maps produced using these threshold values, the Washington State Department of Health guidelines [34] suggest extreme caution with interpretation since the population (denominator) values are less than 100. In fact, the guidelines recommend interpretation with caution for maps produced using populations less than 300. Thus, thresholds, *h *≤ 100 may not be an appropriate choice to use. In this paper, we included values lower than 100 to evaluate the impact of choices that may be considered undesirable. This is also true of *h*_*pi*_ and *h*_*scv*_ for age specific groups in this study. In contrast to these small thresholds, larger thresholds provide more smoothed estimates and will not capture adequate geographic detail on a map. For example, *h* = 5000 and 10,000, may be too large for all age groups since the density curve estimates are almost flat (Fig. 4 column B).

While six thresholds result in similar density curve characteristics for all age groups and may be considered too small (*h* = 50, 100, *h*_*pi*_, and *h*_*scv*_) or too large (*h* = 5000 and 10,000), the remaining thresholds—*h*_*ns*_, *median*, 500, and 1000—provide slightly different density curve characteristics between age 35 years and older and other age groups. For the age groups 35–44, 45–54, 55–64, and 65 years and older, the population density estimates computed from *h* = *h*_*ns*_, *median*, and 500 (arbitrary choice) provide similar density curve characteristics. Thus, when *h* = *h*_*ns*_, the density estimates are smoother and the fluctuations in the tails cease to exist. When threshold values increase (*h* = *median* and 500), the density estimates retain the modal structure of *h* = *h*_*ns*_ but are more smoothed. This density curve characteristic is the most desirable compared to the others as it offers a reasonable compromise between smoothing of the mode and tail.

The thresholds for age 35 years and older that fit this characteristic are *h* = *h*_*ns*_, 500, and 1000. Although thresholds *h* = 500 and *h*_*ns*_ fall in the most desirable characteristic for all age groups, their density curve characteristics between age 35 years and older and other age groups are slightly different. When *h* = 500, the density curve is smoother than *h* = *h*_*ns*_ for the age groups 35–44, 45–54, 55–64, and 65 years and older. In contrast, *h* = *h*_*ns*_ offers a smoother density curve than *h* = 500 for age 35 years and older. Moreover, when *h* = 1000, the resulting density curve retains the same modal structure as *h* = *h*_*ns*_ and 500 (which may be considered as a desirable choice for age 35 years and older), but may be too large for other age groups since the density curves are even more smoother and almost flat. Differences in population size and distribution between age 35 years and older and other age groups are probably the reason. This explanation also applies to *h* = *median*, which may be considered as one of desirable choices for age-specific groups but may be too large threshold for ages 35 years and older.

These findings suggest that thresholds that produce desirable characteristics for one group may not necessarily work for other groups possibly due to differences in population size and distribution. For producing disease maps that incorporate the population age structure, e.g., directly age-adjusted maps, the mapmaker must be careful not to choose different threshold values for each age strata as this could lead to the use of inconsistent spatial supports. Generally, spatial supports must be consistently applied across the entire map [35–37]. In such circumstances, the mapmakers may choose a threshold value that best fits a majority of the age groups.

Table 4 summarizes the characteristics of density curve estimates from various thresholds by age groups. The thresholds that provide the most desirable density curve characteristics are *h* = *h*_*ns*_, *median* and 500 for age groups 35–44, 45–54, 55–64, and 65 years and older and *h* = *h*_*ns*_, 500, and 1000 for age 35 years and older. This is consistent with the recommendation of Silverman [13], to use values that best replicate the population distribution. Additional considerations may include a comparison of the estimated rates, obtained from various threshold estimators, to the actual state rates.

**Table 4:** Characteristics of the population density curve estimates from various thresholds stratified by age groups

Desirable characteristics	Density curve characteristics	Age groups				
		35–44	45–54	55–64	65+	35+
Most ↓ Least	Density curve is smoother, and fluctuations in the tail ceases to exist	*h*_*ns*_	*h*_*ns*_	*h*_*ns*_	*h*_*ns*_	500
		*Median*	*Median*	*Median*	*Median*	*h*_*ns*_
		500	500	500	500	1000
	Density curve closely matches to the actual gamma distribution and contains fluctuations at the tail	100	100	100	100	*h*_*pi*_ *h*_*scv*_
	The highest density estimates of density curve is greater than that of the actual gamma distribution, and the density curve contains high fluctuations at the tail	50	50	50	50	50
		*h*_*pi*_	*h*_*pi*_	*h*_*pi*_	*h*_*pi*_	100
		*h*_*scv*_	*h*_*scv*_	*h*_*scv*_	*h*_*scv*_	
	Density curve is smoother and difficult to distinguish between the mode and tail	1000	1000	1000	1000	*Median*
	Density curve is flat and cannot distinguish between the mode and tail	5000	5000	5000	5000	5000
		10,000	10,000	10,000	10,000	10,000

### Impact of threshold choice on the distribution of rate estimates

**Fig. 5:**
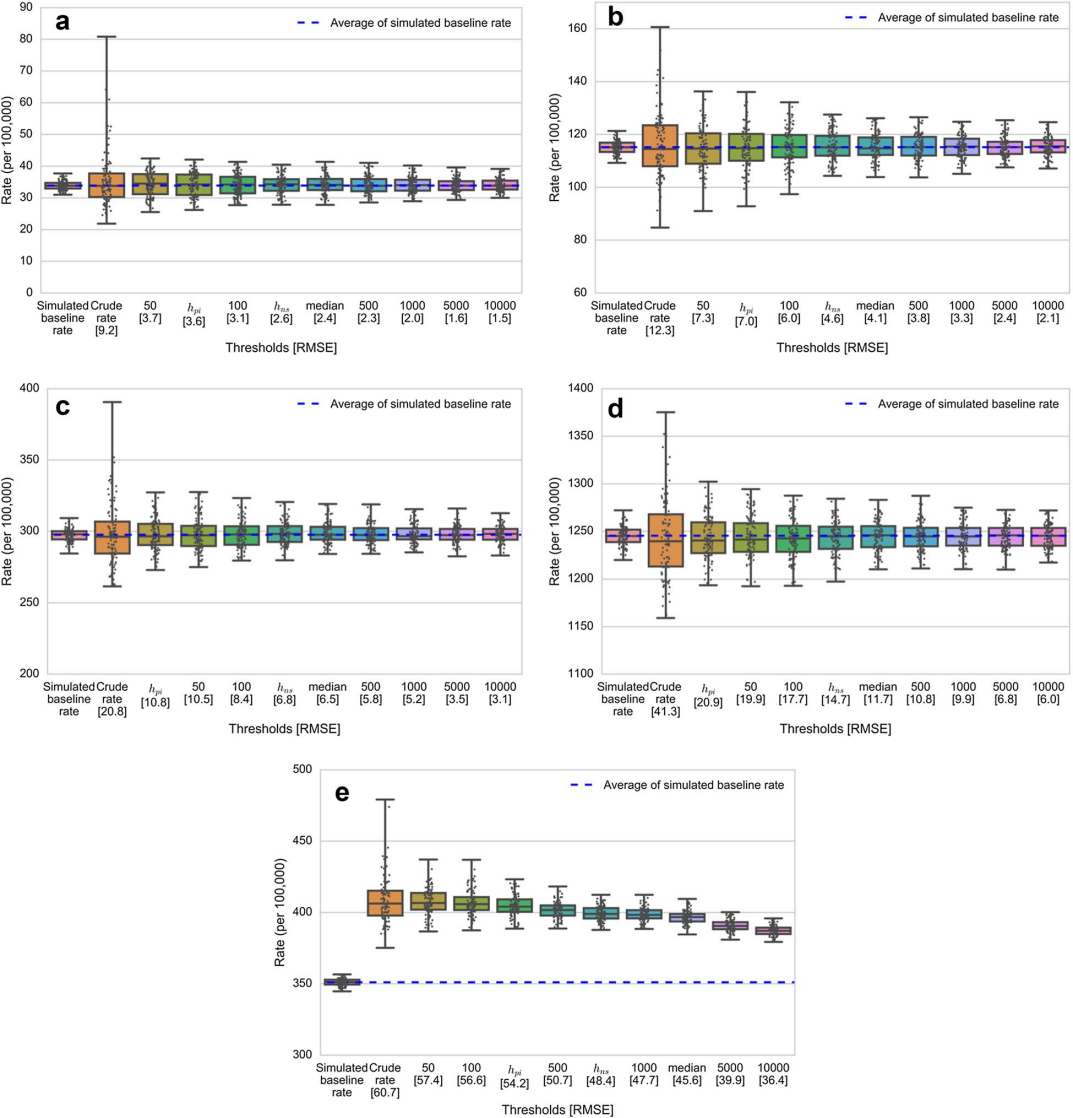
The distribution of estimated state rates of each threshold from 100 repetitions: **a** Aged 35 to 44 years; **b** Aged 45 to 54 years; **c** Aged 55 to 64 years; **d** Aged 65 years and older; **e** Aged 35 years and older

Figure 5 illustrates the distribution of the estimated state rates ($\widehat{y}_{i,l}$) of each threshold from 100 repetitions. Since *h*_*pi*_ and *h*_*scv*_ provided almost identical values for all age groups, only *h*_*pi*_ was used in this study. For each chart, the X-axis represents the thresholds that were used to compute the estimated rates ordered from the smallest to the largest, and the number in the bracket is the RMSE of each threshold (RMSE_*i,t*_ from Eq. 6). The Y-axis shows heart disease mortality rates (per 100,000 population) obtained from the simulated dataset, and each dot represents the estimated state rate for each simulation ($\widehat{y}_{i,l}$). The simulated baseline rate (*y*_*i*_) and the crude rate are also included in each chart for reference. A crude rate was computed as the average of the ratio of simulated cases to population for each individual ZCTA. Note that the scale of the Y-axis is different for each chart—this was done to account for the large differences in heart disease risk between age groups (e.g., the average heart disease death rates for age groups 35–44 and 65 years and older are 33.87 and 1245.93 per 100,000 population, respectively). Also, the crude rate (second boxplot in each panel in Fig. 5) shows greater variation in estimated rates compared to all other boxplots. Moreover, the results show that the variation in rates decreases as thresholds increase. The smaller box plots indicate that the estimated state rates for each map resulting from each simulation tends to be more consistent, and vice versa.

For the age group from 35 to 44 (Fig. 5a), the median rate (the middle line in the boxplot) obtained for each threshold is similar. However, the width of the boxes shrinks towards the center in both the upper and lower quartiles when thresholds increase. This indicates that the estimated state rates are more consistent. Further, the boxplots tend to be similar in structure for thresholds greater than 300 (*h*_*ns*_ ≥* h* ≥1000), in which desirable thresholds are included. The patterns of boxplots in Fig. 5b (45–54 age group), Fig. 5c (55–64 age group), and Fig. 5d (65 years and older) follow similar trends while the boxplots for age group 35 years and older follow slightly different trends (Fig. 5e). Thus, although the overall width of each boxplot decreases with increasing threshold, the median values also decline as thresholds increase.

The inconsistency of estimated state rates for small threshold values is probably due to the small numbers problem, specifically when *h* ≤ 100. This is to be expected since the threshold values (*h*) used to compute the estimated rates in this study are the minimum population size (denominator). Using small threshold values can result in unstable and unreliable rate estimates in spatial units with small population sizes. These unstable rates can affect the estimated state rates since they are aggregated from the smaller spatial units—ZCTA in this study. These results also suggest that *h* ≤ 100 may not be an appropriate choice to use.

### Impact of threshold choice on disease maps

**Fig. 6:**
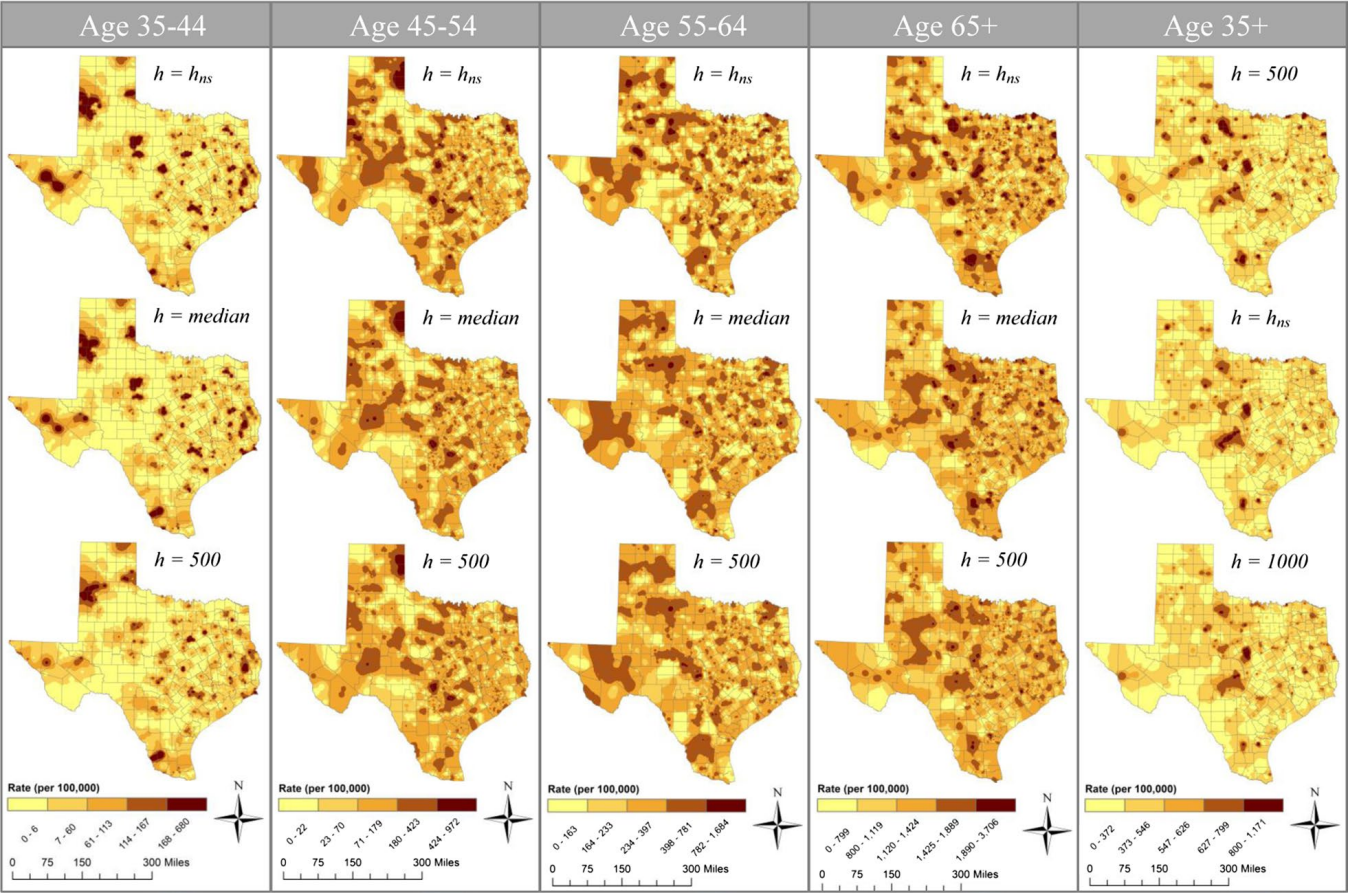
Geographic distribution of age-specific heart disease mortality rates for males aged 35 years and older. Maps were created using the adaptive KDE method with simulated cases as numerators, population data as denominator, and threshold choices (*h*) derived from the bandwidth selector methods and arbitrary choices

As indicated in Table 4, threshold values obtained using *h*_*ns*_, *median*, and *h *>500 provided the most desirable density curve characteristics for the age stratifications used in this study. Further, *h *>500, *h*_*ns*_, and *h *>1000 provided the most desirable density curve characteristics for ages 35 years and older. For these cases, although the RMSE values are not noticeably different (indicated in the x-axis of Fig. 5), differences in boxplot widths, as well as their corresponding IQR, suggest different levels of consistency in average rate estimates (Fig. 5). This is particularly true for the 35+ age group in Fig. 5e. When producing disease maps, there is a need to balance the amount of geographic detail portrayed on the map and accuracy of estimated rates. While the RMSE suggests similar degrees of accuracy between the maps produced using the three desirable thresholds, the remaining key factor to consider in selecting an appropriate threshold is the degree of geographic variation. When the geographic variation is the highest priority, *h*_*ns*_ may be the most desirable threshold choice for all age groups since it provides the greatest variation (more geographic detail) among the candidate thresholds, but still produces accurate rates (Fig. 6). Moreover, compared to arbitrary choices, the *h*_*ns*_ provides a consistent way to estimate the appropriate threshold value.

## Conclusion

Determining the appropriate threshold value is essential for disease mapping because it affects the degree of smoothing that occurs on the map. While the utility of automatic bandwidth selection methods has been studied and discussed for use with non-spatial data, a discussion of their application in disease mapping is limited. In this research, we compare methods for selecting threshold values using existing bandwidth selectors for a synthetic dataset on heart disease mortality among males aged 35 years and older in Texas. The results suggest that *h*_*ns*_ is the most desirable threshold for all age-specific groups and the overall population because it provides greater spatial variation in maps while maintaining accuracy in estimated rates. While this is true only for the simulated case data used in this study, our findings underscore the importance of carefully choosing the threshold values to use in disease mapping. In this paper, we outline how a mapmaker may use automatic bandwidth selection methods to inform decisions about threshold values.
